# Supplementary material for: Insight into the Structural Basis for Dual Nucleic Acid—Recognition by the Scaffold Attachment Factor B2 Protein
Source: Int J Mol Sci. 2023 Feb 7;24(4):3286. doi: 10.3390/ijms24043286 (PMC9958909; doi:10.3390/ijms24043286)
Supplement: Supplementary file 1 [file ijms-24-03286-s001.zip › ijms-2158440-supplementary.pdf]

Supplementary Materials

Insight into the Structural Basis for Dual Nucleic Acid—Recognition by the Scaffold Attachment Factor B2 Protein

Sophie M. Korn [1], Julian Von Ehr [1,2], Karthikeyan Dhamotharan [1], Jan-Niklas Tants [1], Rupert Abele [3] and Andreas Schlundt [1,*]

[1] Institute for Molecular Biosciences and Biomolecular Resonance Center (BMRZ) of Goethe University Frankfurt, Max-von-Laue-Str. 7-9, 60438 Frankfurt, Germany
[2] IMPRS on Cellular Biophysics, Max-von-Laue-Str. 7-9, 60438 Frankfurt, Germany
[3] Institute for Biochemistry, Goethe University Frankfurt, Max-von-Laue-Str. 9, 60438 Frankfurt, Germany
* Correspondence: schlundt@bio.uni-frankfurt.de

This document contains:

- 1 Supplementary Table
- 13 Supplementary Figures

Supplementary Table S1: Overview of DNA oligos of this study used to generate SAFB2 SAP domain variants.

Name	Sequence 5‘ → 3‘	Usage
SAP_R45A_fw	CCGTGCGGAACTTAAAAAGGCGAATCTGGATACCGGTGG	SPRINP[(a)] primers to generate single-point mutant SAP_{R45A}
SAP_R45A_rev	CCACCGGTATCCAGATTCGCCTTTTTAAGTTCCGCACGG	
SAP_K53A/S54A_fw	CGCAATCTGGATACCGGTGGTAACGCAGTTCTGATGGAGCGCCTG	SPRINP[(a)] primers to generate double-point mutant $SAP_{K53A/S54A}$
SAP_K53A/S54A_rev	CAGGCGCTCCATCAGAACTGCTGCGTTACCGGTATCCAGATTGCG	
SAP_S31A_fw	GACTCGTCGGTTAGCGGAGTTGCGCG	SDM[(b)] primers to generate single-point mutant SAP_{S31A}
SAP_S31A_rev	CCCGTTTCGGCCACGCCC	
SAP_T26_fw	ACTCGTCGGTTATCGGAGTTGCGC	SDM[(b)] primers to generate N-terminal shortened $SAP_{26\text{-}70}$
SAP_T26_rev	GCCCTGAAAATAAAGATTCTCAGAACCACTGCC	

(a) Site-directed mutagenesis by SPRINP method, according to Edelheit et al; 2009 [1]. SPRINP, Single-Primer Reactions IN Parallel

(b) Cloning according to manufacturer’s protocol included in NEBaseChanger®, New England Biolabs. SDM, site-directed mutagenesis

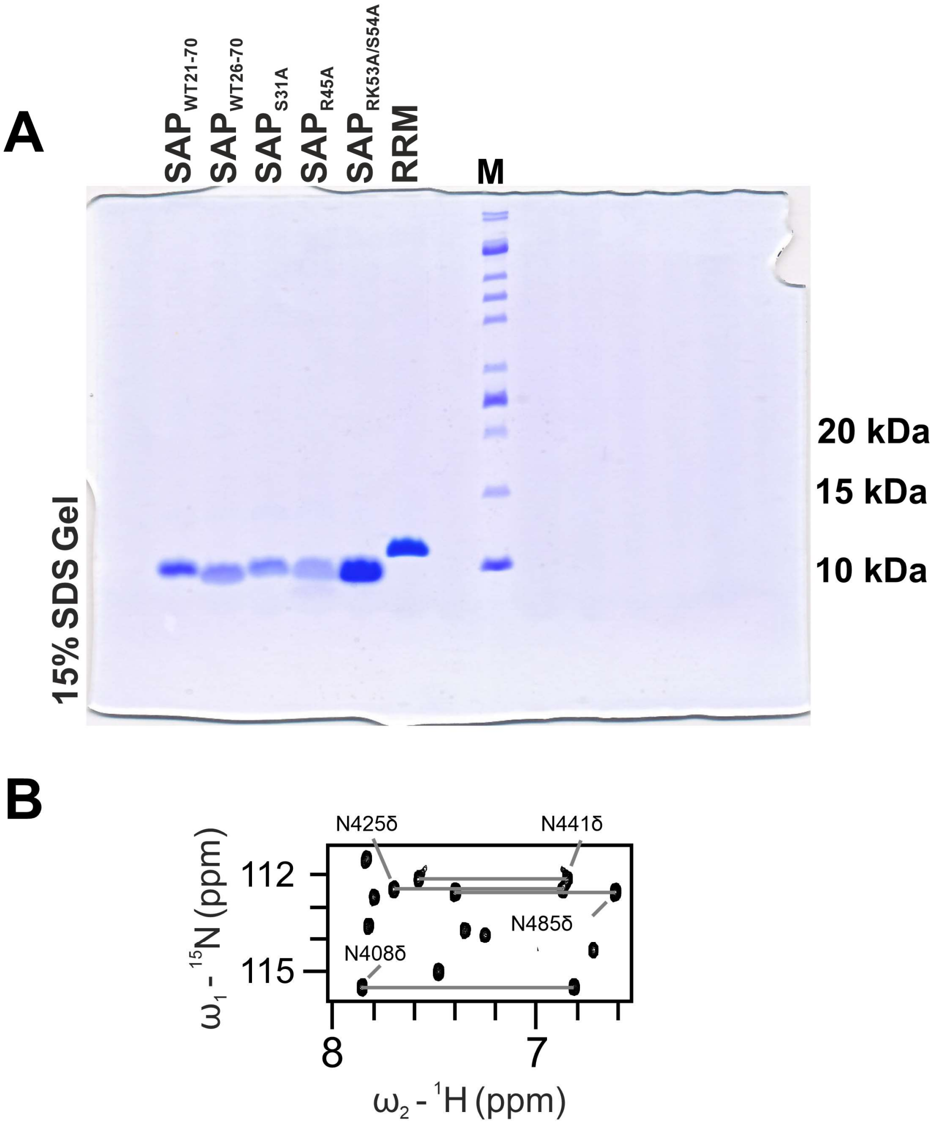

Supplementary Figure S1: (A) Uncropped and unmodified SDS-gel (15% acrylamide) showing representative aliquots of all proteins used in this study (SAP$_{WT21-70}$, SAP$_{WT26-70}$, SAP$_{S31A}$, SAP$_{R45A}$, SAP$_{K53A/S54A}$, and RRM) from final purified samples. **(B)** Zoom-in of the amide sidechain region of the ^{1}H-^{15}N-HSQC of the SAFB2 RRM domain in main text Figure 3. Lines represent pairs of Asn sidechain δ-NH2 groups with their respective assignments given. Assignments of visible backbone amide resonances are, for clarity, shown in main text Figure 3.

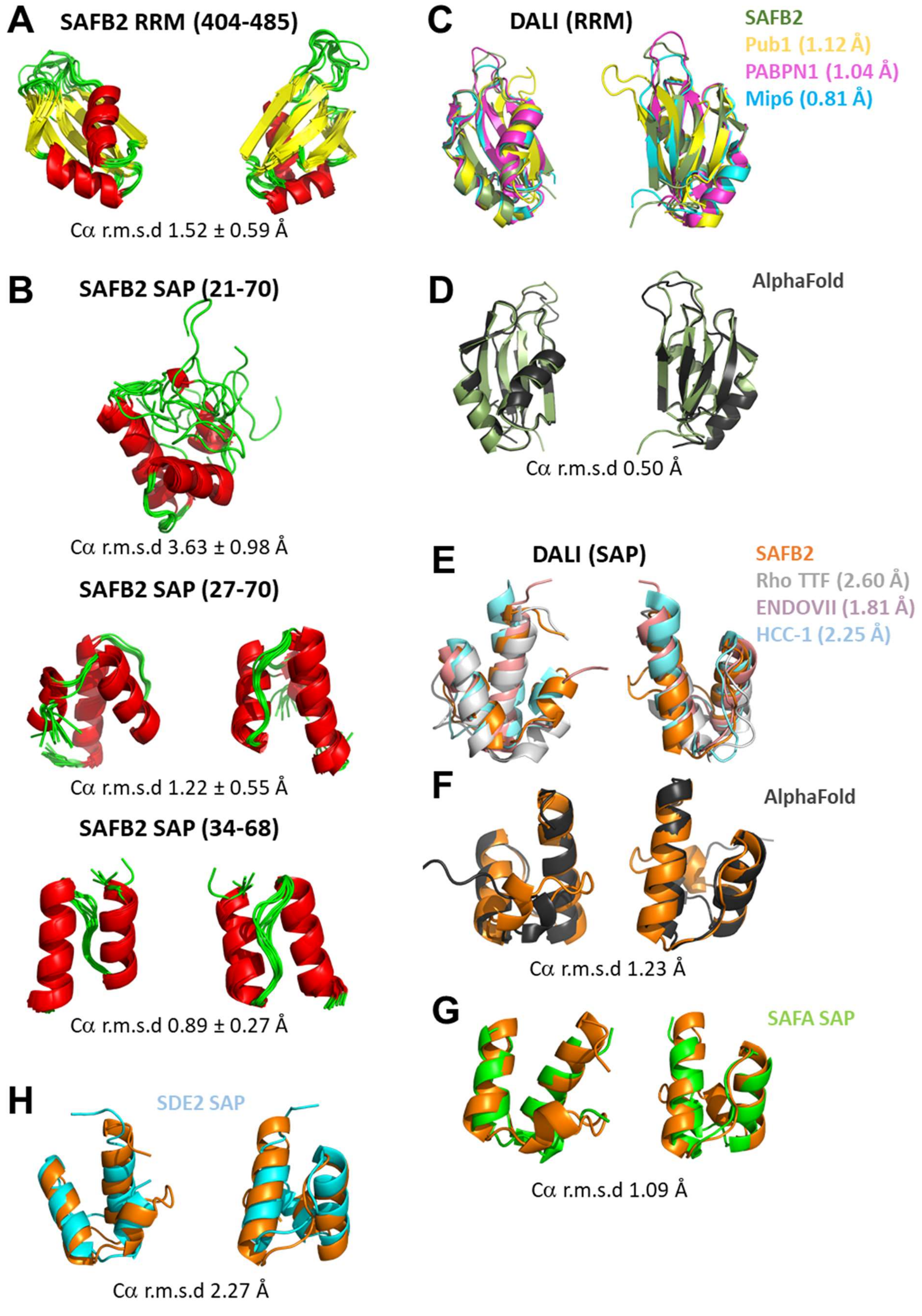

Supplementary Figure S2: CS-Rosetta derived structural models. **(A)** Ensemble of best 10 structures obtained from CS-Rosetta [2] for the SAFB2 RRM. The RMSD value to the lowest energy structure is given as average and standard deviation. **(B)** The same as

in panel A for the SAFB2 SAP domain. The ensemble is shown as structural alignment for the full NMR construct (top), the N-terminally truncated version (middle, see also Suppl. Fig. 3) and the two-helical core domain (bottom), indicating increasing convergence. **(C, D)** Comparison of the CS-Rosetta-derived lowest energy SAFB2 RRM domain structure with the three best hits obtained from a DALI [3] search (C) or with an AlphaFold-derived [4] model of the identical boundaries (D). Individual rmsd values are given. Underlying PDB codes are: 3MD3 (Pub1, unpublished), 3B4M (PABPN1 [5]), 5D77 (Mip6, unpublished). **(E-H)** Comparison of the CS-Rosetta-derived lowest energy SAFB2 SAP domain structure with either the three best hits obtained from a DALI [3] search (E), with an AlphaFold-derived model of the identical boundaries (F), with the SAP domain of SAFA, or with the SAP domain of ssDNA-binding SDE2 using analogous boundaries (G). Individual rmsd values are given. Underlying PDB codes are: 5JJI (Rho TTF [6]), 1E7D (ENDOVII [7]), 7N99 (SDE2) [8], 2DO1 (HCC-1, unpublished).

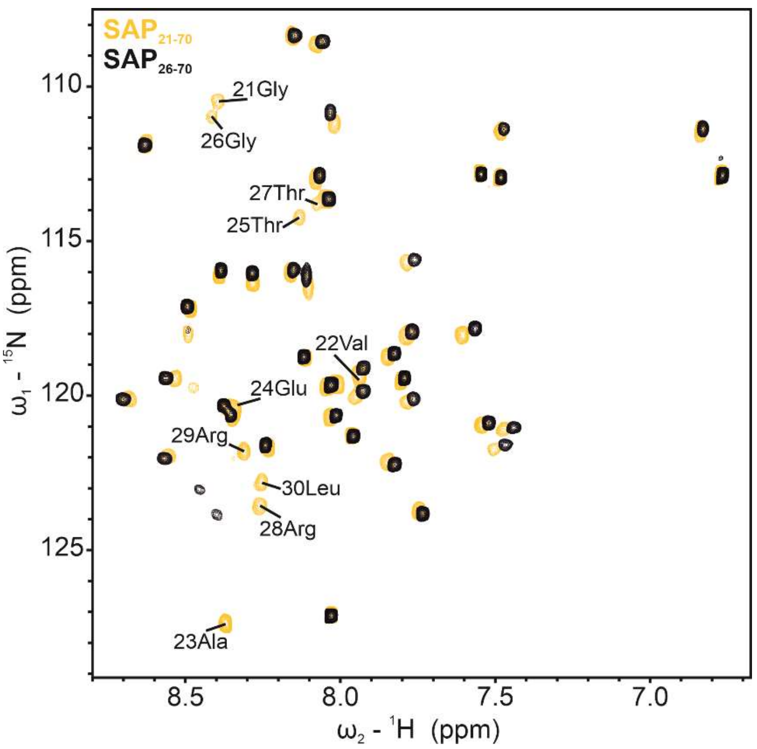

Supplementary Figure S3: N-terminal truncation of SAFB2 SAP does not affect the domain's core fold. Overlay of ^{1}H-^{15}N-HSQC spectra of wild-type SAP_{21-70} (yellow) and SAP_{26-70} (black). N-terminal residues are labelled with their assignments for orientation. The new Gly26 amide of SAP_{26-70} is line-broadened beyond detection. Only residues of the N-terminal extension within the truncated and non-truncated SAP versions are affected with respect to each other as seen by their perturbed chemical shifts.

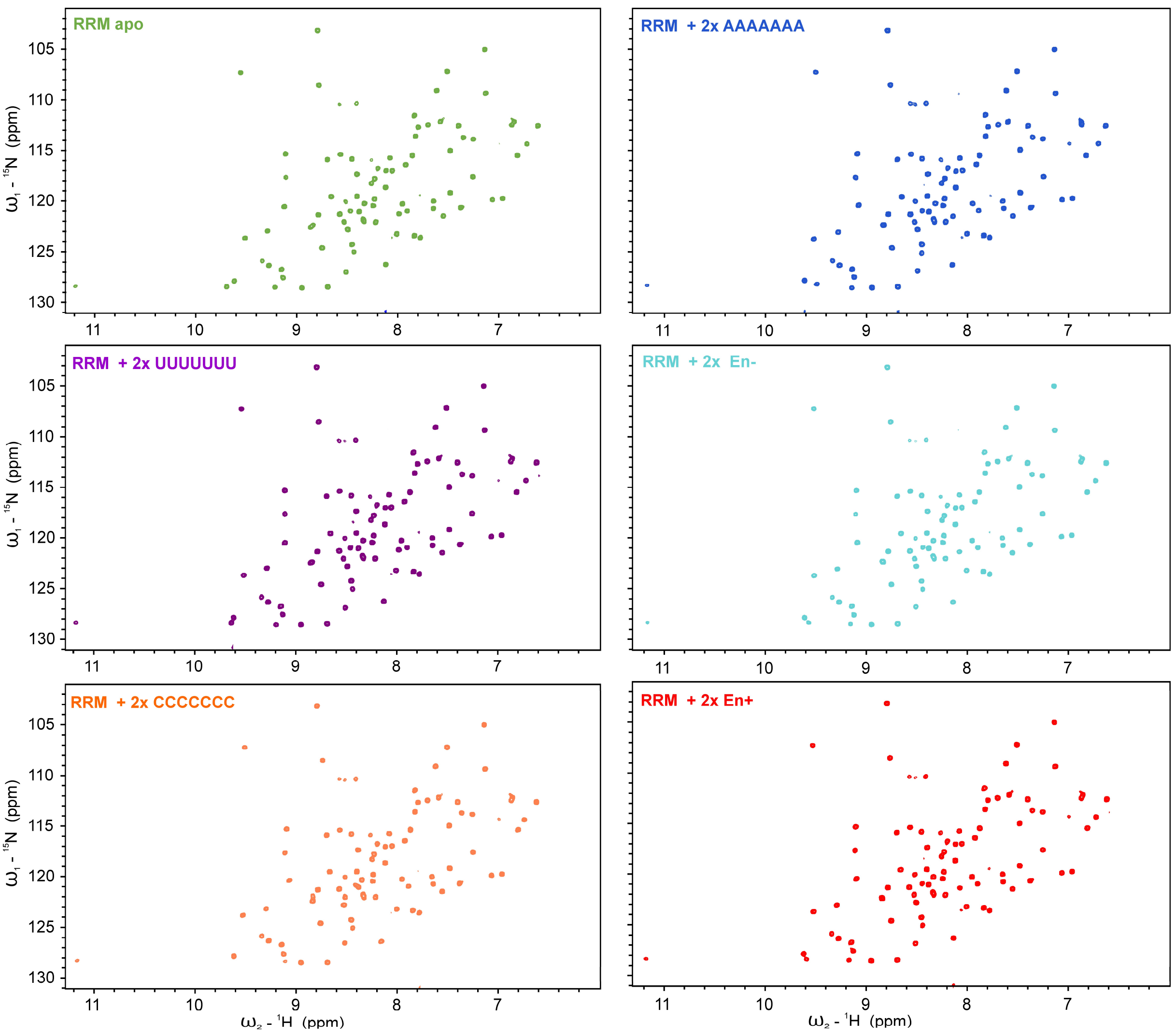

Supplementary Figure S4: RNA preferences of the SAFB2 RRM domain. Full ^{1}H-^{15}N HSQC spectra of the apo RRM (green, top left) and the RRM after addition of 2x molar excess of either U_7 (purple, middle left), C_7 (orange, bottom left), A_7 (blue, top right), En+ (cyan, middle right) or En- (red, bottom right). Spectra correspond to zoom-ins in main text Figure 4.

A

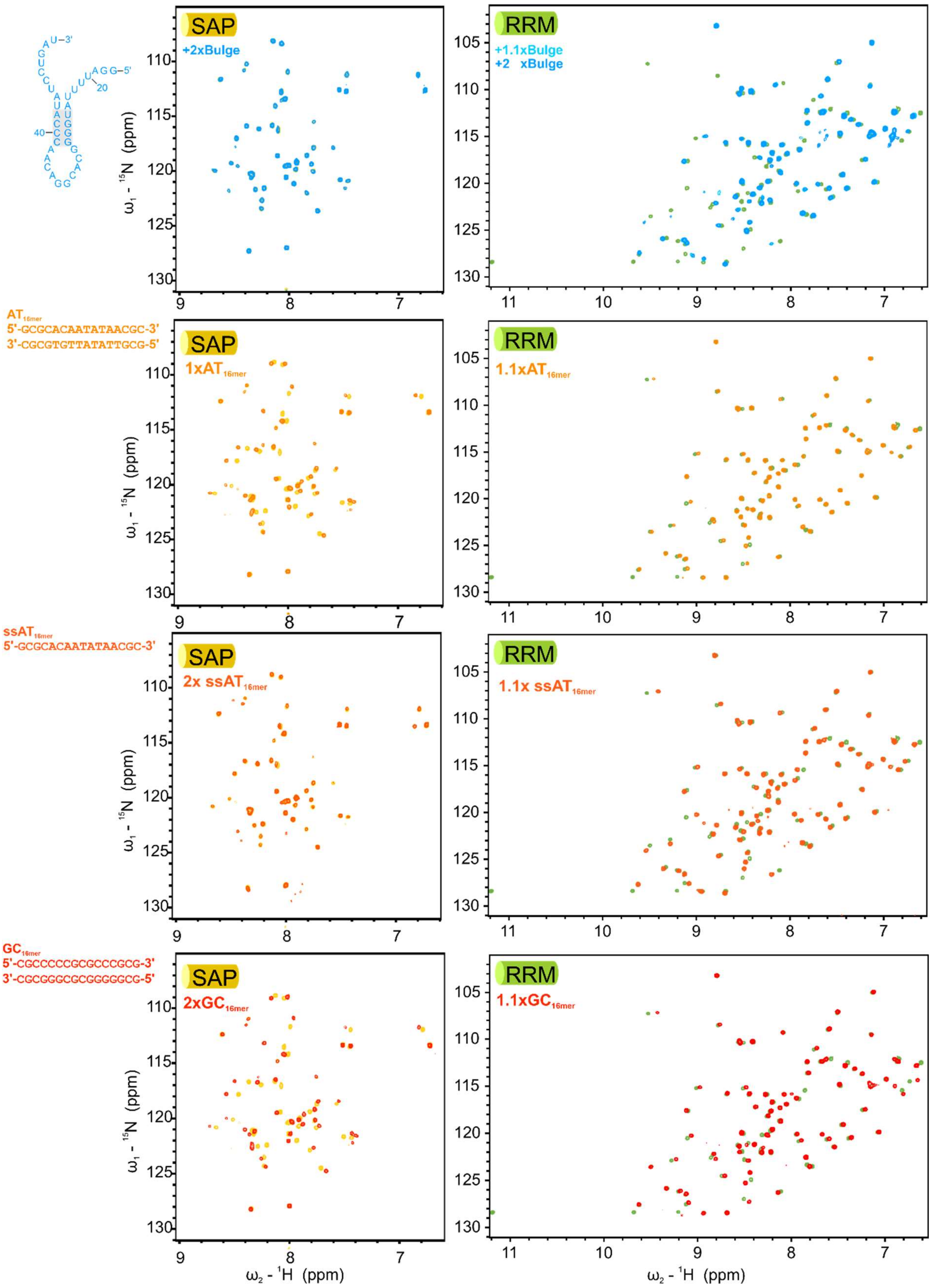

SAP
+2xBulge
RRM
+1.1xBulge
+2 xBulge
AT16mer
5'-GCGCACAATATAACGC-3'
3'-CGCGTGTTATATTGCG-5'
1xAT16mer
1.1xAT16mer
ssAT16mer
5'-GCGCACAATATAACGC-3'
2x ssAT16mer
1.1x ssAT16mer
GC16mer
5'-CGCCCCCGCGCCCGCG-3'
3'-CGCGGGCGCGGGGGCG-5'
2xGC16mer
1.1xGC16mer
ω1 - 15N (ppm)
ω2 - 1H (ppm)

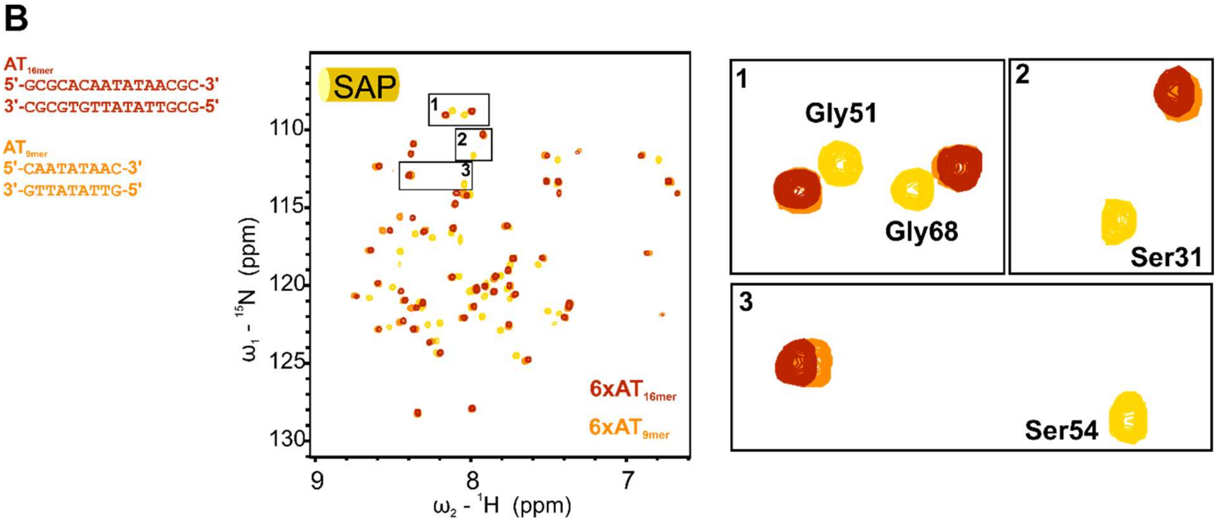

Supplementary Figure S5: Binding preferences of the SAFB2 SAP and RRM domains for different nucleic acid types. **(A)** Overlays of full ^{1}H-^{15}N HSQC spectra of the apo SAP domain (left column) or the RRM domain (right column) with spectra of the proteins in complex with respective molar excess (as indicated) of either Bulge RNA (purple, middle left), dsAT$_{16mer}$, ssAT$_{16mer}$ or dsGC$_{16mer}$ (from top to bottom). **(B)** Overlay of the full ^{1}H-^{15}N HSQC apo SAP spectrum with those of SAP in presence of molar excess of dsAT$_{16mer}$ (red) and dsAT$_{9mer}$ (orange). Zoom-ins of HSQC regions 1, 2 and 3 (right) reveal identical CSP trajectories for the 9mer and the 16mer dsDNA indicating the same binding mode.

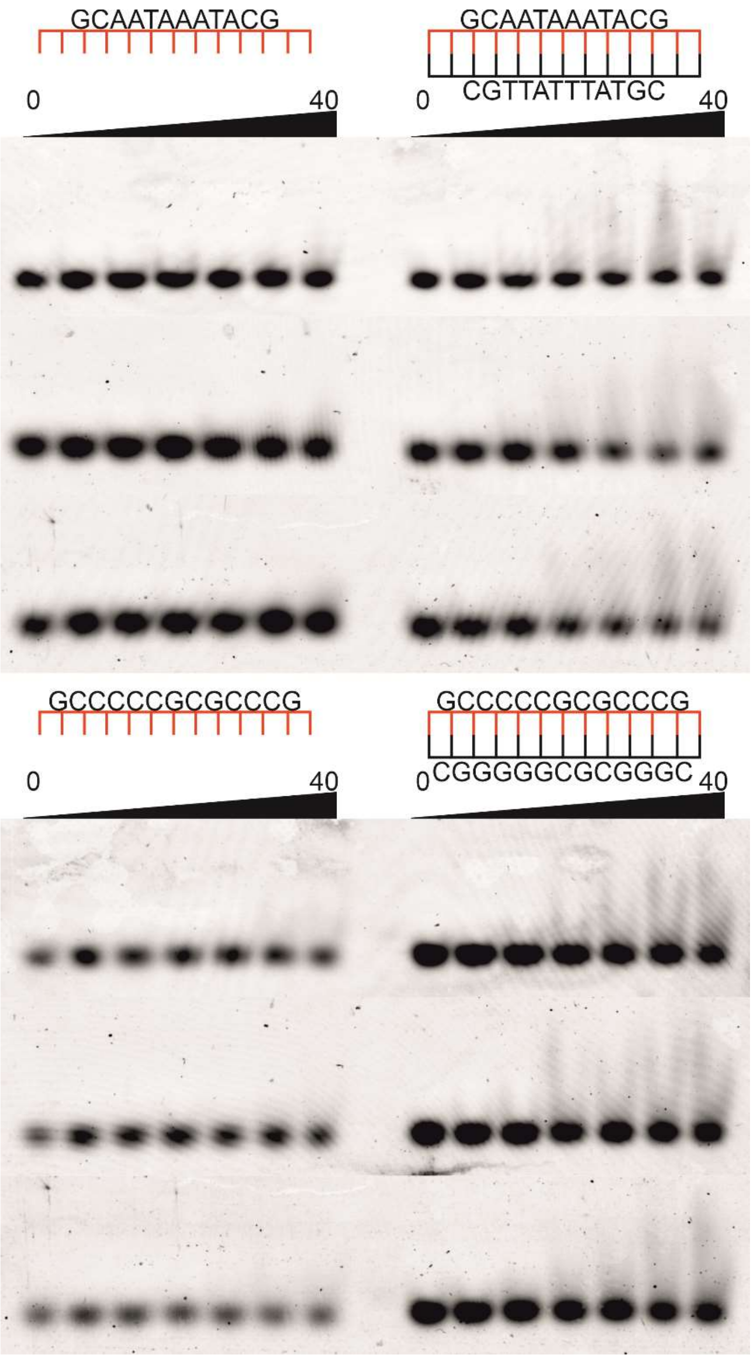

Supplementary Figure S6: Depiction of DNAs in EMSA triplicates of the SAFB2 SAP domain titrated to single-stranded (left) and double-stranded (right) 13mer AT-rich or GC-rich DNAs (top and bottom, respectively) corresponding to main text Figure 5. Increasing protein concentrations (0-40 μM) are indicated by black bars above gel pictures.

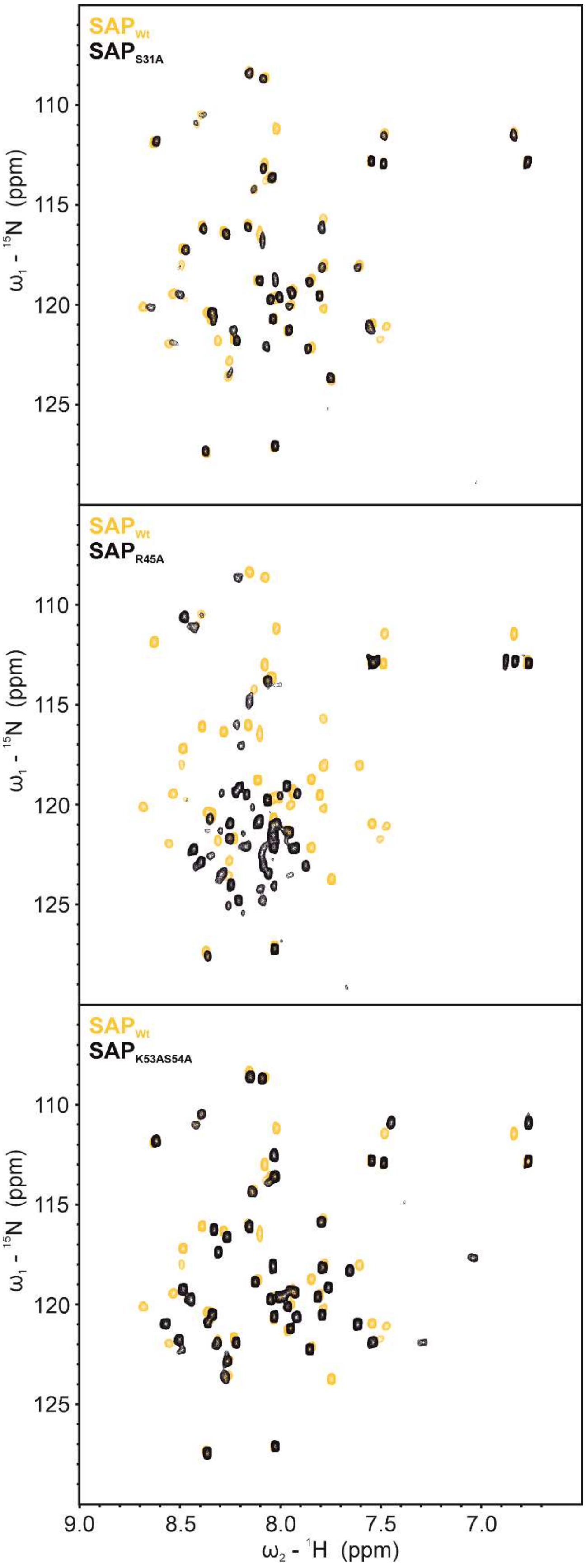

Supplementary Figure S7: Effects of individual point mutations on the SAFB2 SAP domain fold. Full ^{1}H-^{15}N HSQC spectra of mutant proteins: SAP$_{S31A}$, SAP$_{R45A}$ and SAP$_{K53AS54A}$ (from top to bottom), each overlaid with SAP$_{WT}$ for comparison.

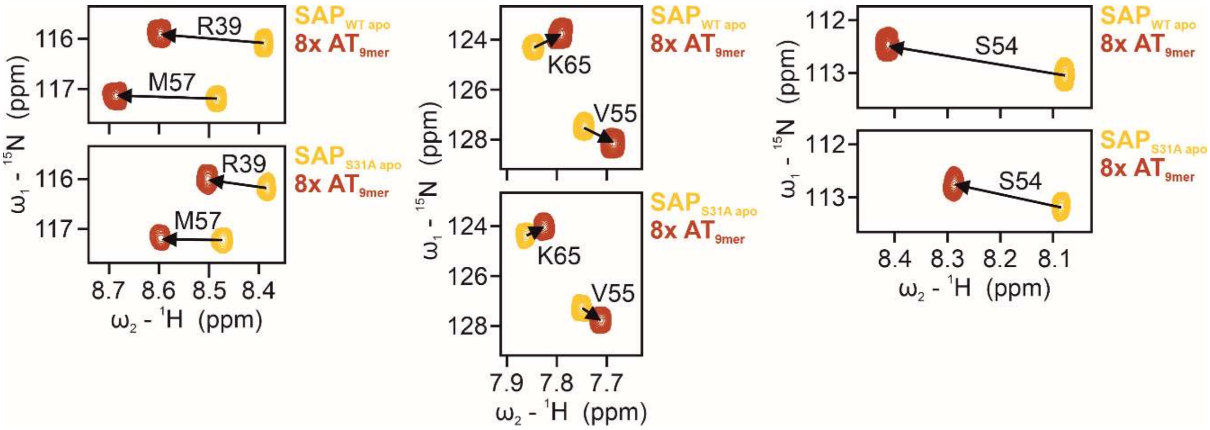

Supplementary Figure S8: Effect of the S31A mutation in the SAFB2 SAP domain on DNA binding. Identical insets of ^{1}H-^{15}N HSQC spectra of the SAP_{WT} (upper panels) and the mutant SAP_{S31A} (lower panels). Each overlay shows the apo protein (yellow) vs. complexes with 8- fold excess of AT_{9mer} (red). Arrows indicate their respective CSP trajectories (for the selected residues). While trajectories are the same, the S31A mutant displays significantly reduced CSP extents. Consequently, the mutation reduces the affinity of SAP for the DNAs, but does not alter the mode of interaction or prohibit complex formation.

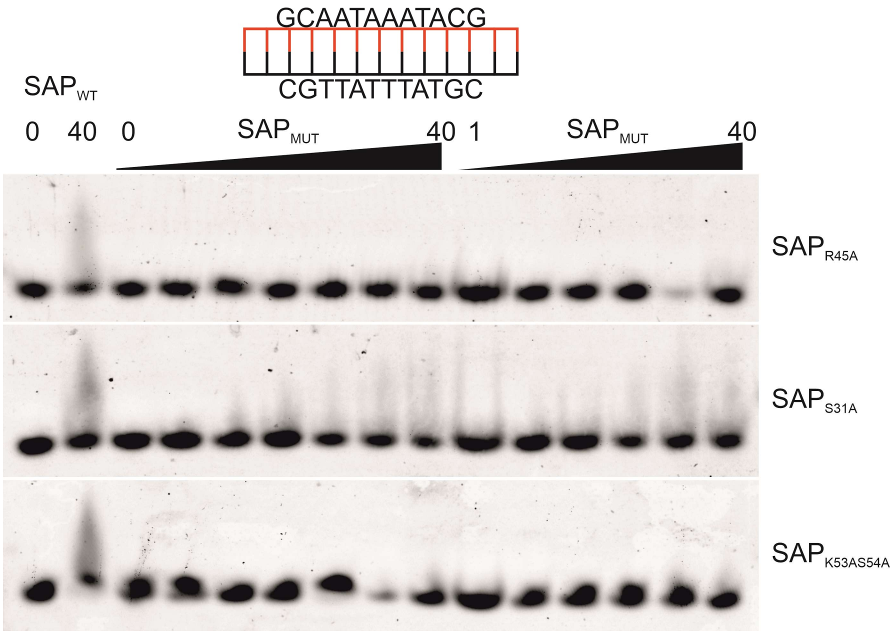

Supplementary Figure S9: DNA-binding capacity of SAFB2 SAP domain mutants. Depiction of DNA in EMSA duplicates of the SAFB2 SAP domain mutants as given when titrated to 13mer AT-rich DNA. Increasing protein concentrations of mutants (0/1-40 μM) are indicated by black bars above gel pictures. The second lane from left shows SAP$_{WT}$ at 40 μM as a reference. In line with data from NMR, the EMSAs show no binding of the mutants R45A and KS5354AA, but only slightly impaired binding in the S31A mutant.

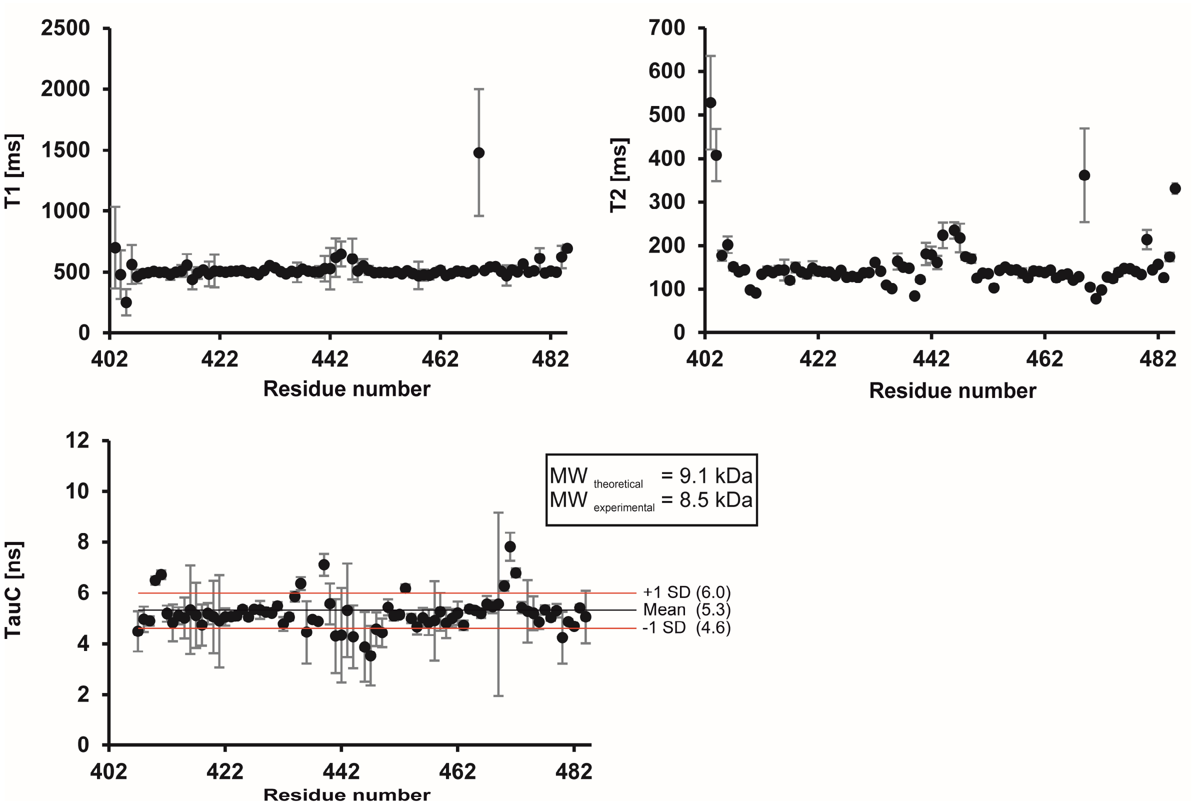

Supplementary Figure S10: ^{15}N relaxation data for amides of the SAFB2 RRM domain. The upper two panels show ^{15}N T1 and T2 times as a function of SAFB2 RRM domain amides recorded at an Avance II Bruker spectrometer (600 MHz proton Larmor frequency) and 298 K. For T1 and T2 all values are shown. Rotational correlation times TauC in the lower panel are derived from T1 and T2, but were calculated only for rigid parts according to hetNOE and SCS (see main text Figure 4) values, and are shown with mean value ± 1 SD. T1 and T2 errors are determined by the program *CCPNMR Analysis* [9]; TauC errors are the sum of percental errors obtained for T1 and T2.

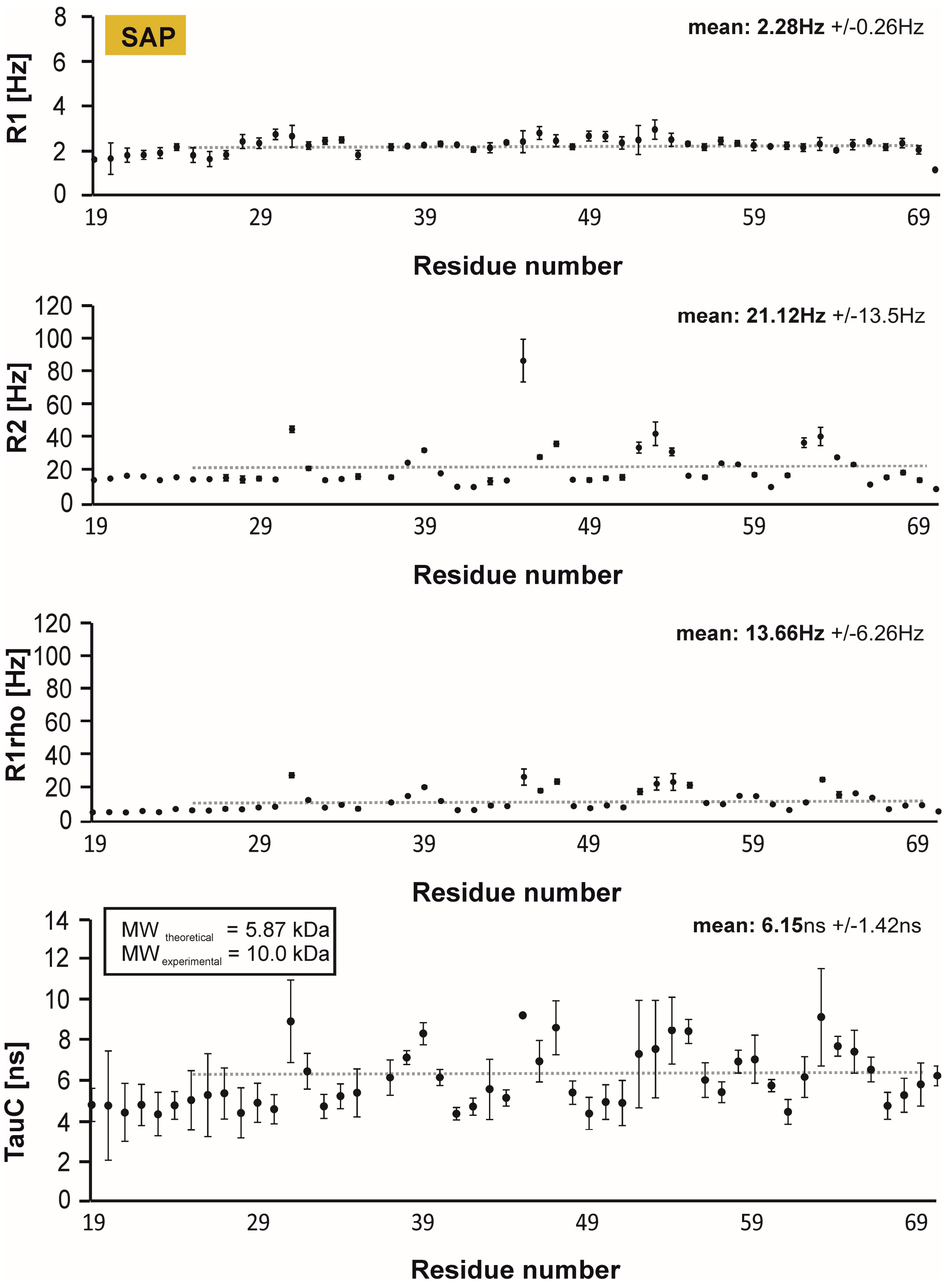

Supplementary Figure S11: ^{15}N relaxation data for the SAFB2 SAP$_{WT}$. R1, R2 and R1rho rates were determined from measurements at 700 MHz (proton Larmor frequency) and 298 K. Mean values were calculated for the structured domain indicated by grey, dotted lines, respectively. Errors for relaxation rates are derived from calculations using *CCPNMR Analysis* [9]. Mean TauC values (rotational correlation times) were used to estimate the SAP domain molecular weight (MW$_{experimental}$) and compared to the theoretical MW$_{theoretical}$ (as given in the box). Errors for TauC are the sum of percental errors obtained for R1 and R1rho.

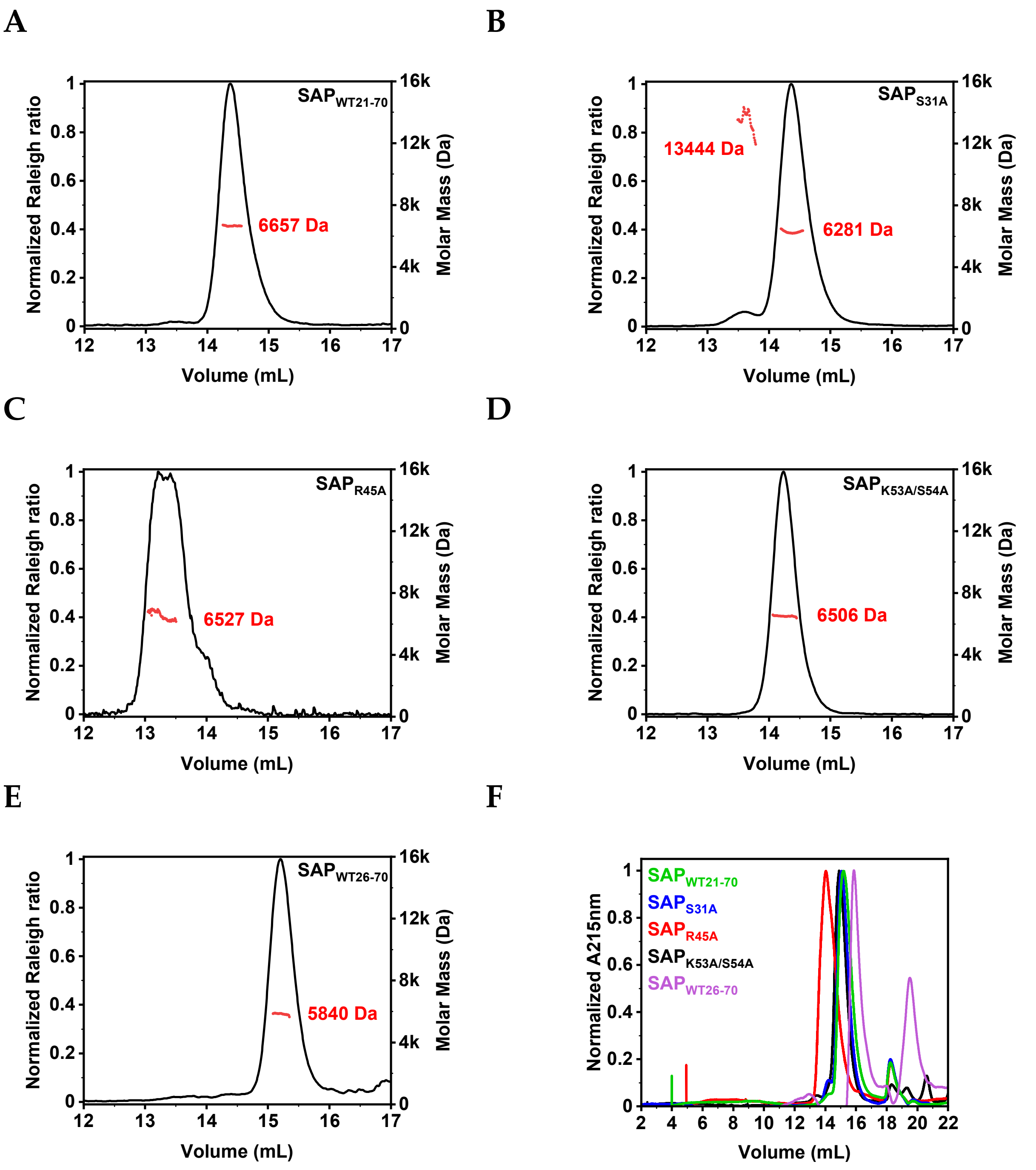

G

	SAP$_{WT21-70}$	SAP$_{31A}$	SAP$_{R45A}$	SAP$_{K53A/S54A}$	SAP$_{WT26-70}$
Theoretical MW (Da)	5869	5853	5784	5796	5152
aSEC based MW (Da)	9810	10360	14857	10801	7620

Supplementary Figure S12: Analytical SEC and SEC-MALS data on SAP wild-type and mutants. **(A-E)** SEC-MALS analysis of SAP$_{WT21-70}$, SAP$_{31A}$, SAP$_{R45A}$, SAP$_{K53A/S54A}$ and SAP$_{WT26-70}$, respectively. Shown are the SEC chromatograms with the peak of interest and the MW as derived from the intersected peak along the elution volume as indicated by the red trace. **(F)** Analytical SEC of SAP wild-type and mutants. Full chromatograms corresponding to main text Figure 7. **(G)** Molecular weight estimation

of SAP wild-type and mutants derived from a calibration curve based on elution volumes of MW standards when compared to the theoretical MWs of the respective monomers. All SEC runs in this figure were carried out on a SD75 Increase 10/300 GL column.

A

B

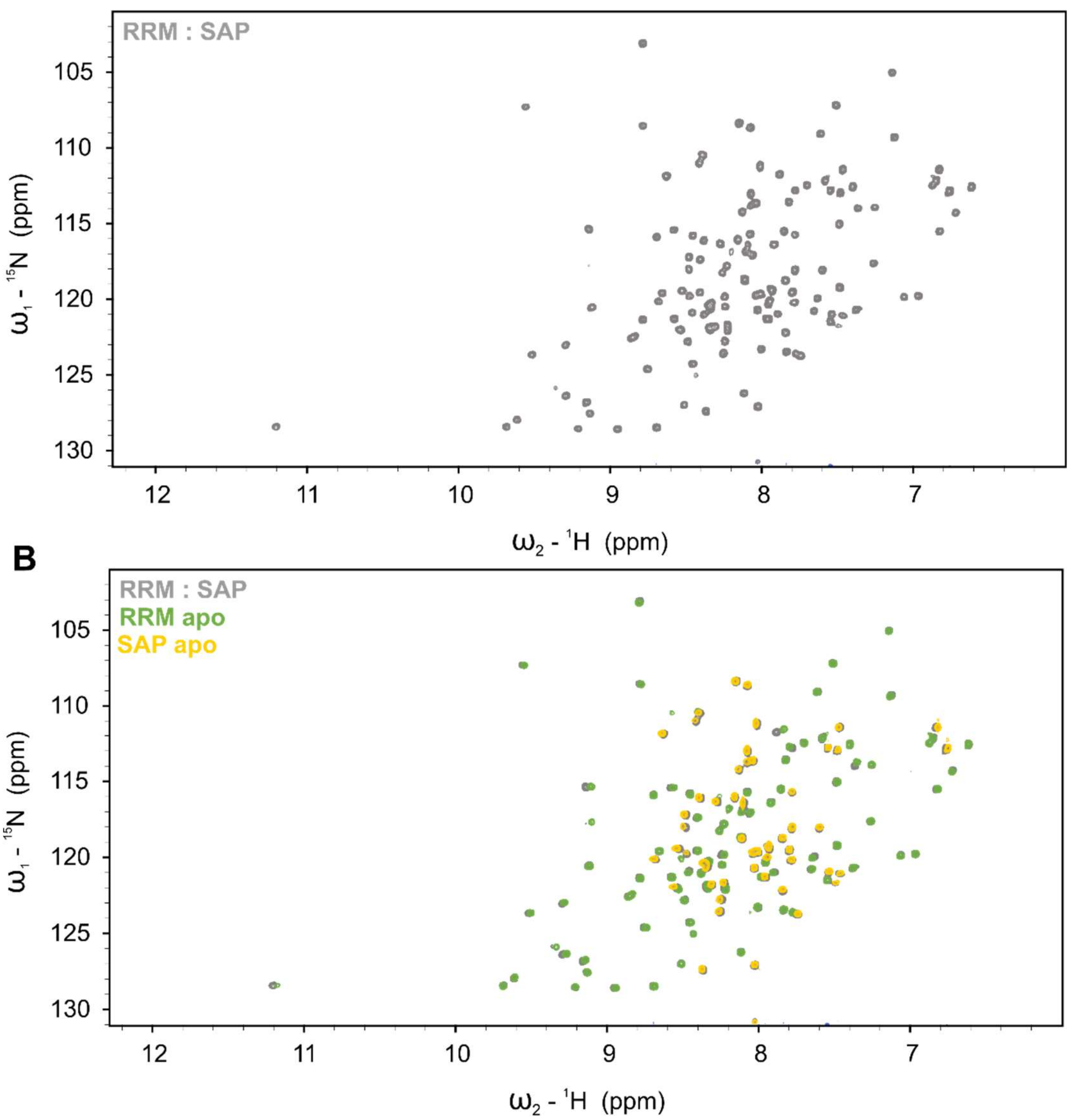

Supplementary Figure S13: The SAFB2 SAP and RRM domains do not interact. **(A)** ^{1}H-^{15}N HSQC of a mixed sample of ^{15}N-labelled SAP and ^{15}N-labelled RRM domains in a 1:1 ratio. **(B)** Overlay of the ^{1}H-^{15}N HSQC spectra of the 1:1 SAP/RRM mix from panel A with the respective spectra of individual ^{15}N-labelled domains (RRM – green; SAP – yellow). The unperturbed peak patterns of either domain in the mixture indicate the two domains to be independent from each other.

Supplementary references:

1. Edelheit, O.; Hanukoglu, A.; Hanukoglu, I. Simple and efficient site-directed mutagenesis using two single-primer reactions in parallel to generate mutants for protein structure-function studies. *BMC Biotechnol* **2009**, *9*, 61, doi:10.1186/1472-6750-9-61.
2. Lange, O.F.; Rossi, P.; Sgourakis, N.G.; Song, Y.; Lee, H.W.; Aramini, J.M.; Ertekin, A.; Xiao, R.; Acton, T.B.; Montelione, G.T.; et al. Determination of solution structures of proteins up to 40 kDa using CS-Rosetta with sparse NMR data from deuterated samples. *Proc Natl Acad Sci U S A* **2012**, *109*, 10873-10878, doi:10.1073/pnas.1203013109.
3. Holm, L.; Rosenstrom, P. Dali server: conservation mapping in 3D. *Nucleic Acids Res* **2010**, *38*, W545-549, doi:10.1093/nar/gkq366.
4. Jumper, J.; Evans, R.; Pritzel, A.; Green, T.; Figurnov, M.; Ronneberger, O.; Tunyasuvunakool, K.; Bates, R.; Zidek, A.; Potapenko, A.; et al. Highly accurate protein structure prediction with AlphaFold. *Nature* **2021**, *596*, 583-589, doi:10.1038/s41586-021-03819-2.
5. Ge, H.; Zhou, D.; Tong, S.; Gao, Y.; Teng, M.; Niu, L. Crystal structure and possible dimerization of the single RRM of human PABPN1. *Proteins* **2008**, *71*, 1539-1545, doi:10.1002/prot.21973.
6. Thomsen, N.D.; Lawson, M.R.; Witkowsky, L.B.; Qu, S.; Berger, J.M. Molecular mechanisms of substrate-controlled ring dynamics and substepping in a nucleic acid-dependent hexameric motor. *Proc Natl Acad Sci U S A* **2016**, *113*, E7691-E7700, doi:10.1073/pnas.1616745113.
7. Raaijmakers, H.; Toro, I.; Birkenbihl, R.; Kemper, B.; Suck, D. Conformational flexibility in T4 endonuclease VII revealed by crystallography: implications for substrate binding and cleavage. *J Mol Biol* **2001**, *308*, 311-323, doi:10.1006/jmbi.2001.4592.
8. Weinheimer, A.S.; Paung, Y.; Rageul, J.; Khan, A.; Lo, N.; Ho, B.; Tong, M.; Alphonse, S.; Seeliger, M.A.; Kim, H. Extended DNA-binding interfaces beyond the canonical SAP domain contribute to the function of replication stress regulator SDE2 at DNA replication forks. *J Biol Chem* **2022**, *298*, 102268, doi:10.1016/j.jbc.2022.102268.
9. Vranken, W.F.; Boucher, W.; Stevens, T.J.; Fogh, R.H.; Pajon, A.; Llinas, P.; Ulrich, E.L.; Markley, J.L.; Ionides, J.; Laue, E.D. The CCPN data model for NMR spectroscopy: Development of a software pipeline. *Proteins-Structure Function and Bioinformatics* **2005**, *59*, 687-696.
